# Acute Mitral Regurgitaion Initially Misdiagnosed As Pneumonia: A Case Report

**DOI:** 10.7759/cureus.74531

**Published:** 2024-11-26

**Authors:** Yoshito Sugi, Kazuaki Nishida, Tetsuro Ishida, Go Inoue, Takushi Fujimoto

**Affiliations:** 1 Department of Emergency and Critical Care, Osaka City General Hospital, Osaka, JPN; 2 Department of Emergency and General Medicine, Mimihara General Hospital, Osaka, JPN; 3 Department of Emergency Medicine, Yodogawa Christian Hospital, Osaka, JPN

**Keywords:** acute mitral regurgitation, cardiorespiratory failure, diagnosis, echocardiography, heart failure

## Abstract

Heart failure due to acute mitral regurgitation (MR) may present with atypical findings on physical examination and transthoracic echocardiography (TTE), making the diagnosis difficult. A 72-year-old male was transferred to our hospital with severe cardiorespiratory failure and was initially suspected to have severe pneumonia based on the acute onset and imaging findings. The patient was eventually diagnosed with acute heart failure secondary to MR. Acute MR can be a fatal disease; clinicians should consider it in the setting of severe respiratory and circulatory failure as it can mimic severe pneumonia and acute respiratory distress syndrome (ARDS).

## Introduction

Acute mitral regurgitation (MR) is a medical and surgical emergency. Patients usually present with severe respiratory and circulatory failure [1]. However, acute MR is often misdiagnosed in the emergency department as acute pulmonary disease because of severe dyspnea, an abnormal chest radiograph, the presence of atypical findings on physical and echocardiographic examinations, and the acute onset [2]. Acute MR is typically ‘silent’ in severely deteriorated hemodynamic conditions because of the equalization of left ventricular and left atrial pressures in mid-systole, leading to a soft or absent murmur and absence of the reflex jet on echocardiography [3]. In the emergency department, any patient with acute respiratory failure and hemodynamic deterioration should have acute heart failure considered in the differential diagnosis, alongside pneumonia and acute respiratory distress syndrome (ARDS). Once heart failure is diagnosed, acute valvular abnormalities, including MR, should be investigated as potential causes. Timely recognition and treatment of acute MR are essential to prevent serious complications like heart failure and shock.

## Case presentation

A 72-year-old male was transferred to our hospital with worsening dyspnea since the previous day. He had no abnormalities noted during medical examinations and was not regularly taking oral medications. He had annual medical check-ups, but nothing unusual was ever noted. He had a smoking history of 30 pack-years, but he ceased 20 years previously and reported low alcohol intake. He had daily contact with his grandchildren aged six, five, four, and one year, but none had recent illnesses.

On the evening prior to presentation at our hospital, he experienced dyspnea without any specific trigger accompanied by a dry cough, which occurred upon returning home after completing his daily fieldwork. He was awakened at 4 am due to worsening dyspnea. 

Physical examination performed upon arrival showed that he was afebrile and alert, with a blood pressure of 97/54 mmHg, pulse rate of 100 beats per minute, respiratory rate of 26 breaths per minute, and oxygen saturation of 85% on 10 L/min of oxygen via a face mask. Accessory sternocleidomastoid muscles were used, the external jugular vein was distended, and chest auscultation revealed crackles over bilateral lung fields. Peripheral coldness or edema of the legs was not observed. The initial arterial blood gas analysis revealed prominent hypoxemia and additional tests later suggested elevated BNP without any elevated myocardial deviation enzymes (Table 1).

**Table 1:** Laboratory data of the patient PaCO_2_: arterial partial pressure of carbon dioxide; PaO_2_: arterial oxygen pressure; BE: base excess; Lac: lactate; WBC: white blood cell count; Neutro: neutrophil; Eosino: eosinophil; Baso: basophil; Mono: monocyte; Lymph: lymphocyte; RBC: red blood cell count; Hb: hemoglobin; Ht: hematocrit; MCV: mean corpuscular volume; MCH: mean corpuscular hemoglobin; MCHC: mean corpuscular hemoglobin concentration; Plt: platelet count; APTT: activated partial thromboplastin time; PT-INR: prothrombin time-international normalized ratio; Glu: glucose; HbA1c: hemoglobin A1c; TP: total protein; Alb: albumin; AST: aspartate aminotransferase; ALT: alanine transaminase; ALP: alkaline phosphatase; LDH: lactate dehydrogenase; CK: creatine kinase; CK-MB: creatine kinase-myocardial band; T-Bil: total bilirubin; BUN: blood urea nitrogen; Cre: creatinine; eGFR: estimated glomerular filtration rate; Na: sodium; K: potassium; Cl: chloride; Ca: calcium; IP: inorganic phosphorus; Mg: magnesium; CRP: C-peptide immunoreactivity; TSH: thyroid-stimulating hormone; FT4: free thyroxine; BNP: brain natriuretic peptide

Test	Result	Units	Reference range
pH	7.483		7.36-7.44
PaCO_2_	45.4	mmHg	32-48
PaO_2_	27.8	mmHg	83-108
HCO_3_^-^	20.8	mEq/L	22-26
BE	-1.3	mEq/L	–2-＋2
Lac	1.9	mmol/L	0.44–1.78
WBC count	9000	/μL	40–80
Neutro	83.4	%	30–70
Eosino	0.1	%	~5.0
Baso	0.3	%	~2.0
Mono	5.9	%	3.0–11.0
Lymph	10.3	%	20.0–50.0
RBC count	424	×10^4^/μL	430–570
Hb	14	g/μL	14.0–17.0
Ht	41.3	%	42.0–54.0
MCV	97.4	Fl	85.0–100.0
MCH	33	pg	27.0–34.0
MCHC	33.9	g/dL	31.0–36.5
PLT count	21.1	×10^4^	15.0–35.0
APTT	30	Seconds	25–40
PT-INR	0.98	Seconds	
Glu	141	mg/dL	70–109
HbA1c	5.3	%	4.6–6.2
TP	6.3	g/dL	6.7–8.3
Alb	3.5	g/dL	3.8–5.3
AST	24	IU/L	9–35
ALT	12	IU/L	5–33
ALP	68	U/L	38–113
LDH	175	U/L	124–222
CK	175	IU/L	57–197
CK-MB	15	IU/L	0–25
T-Bil	1	mg/dL	0.2–1.2
BUN	20.6	mg/dL	6.0–22.0
Cre	1.06	mg/dL	0.50–1.20
eGFR	53.1	ml/min/1.73^2^	60~
Na	140	mmol/L	136–148
K	3.7	mmol/L	3.5–5.0
Cl	110	mmol/L	96–108
Ca	9.2	mg/dL	8.6–10.8
IP	3.1	mg/dL	2.4–4.8
CRP	0.16	mg/dL	0.00–0.30
TSH	2.709	μIU/mL	0.610–4.230
FT4	1.04	ng/dL	0.70–1.70
BNP	516.1	pg/mL	0–18.4

**Figure 1:**
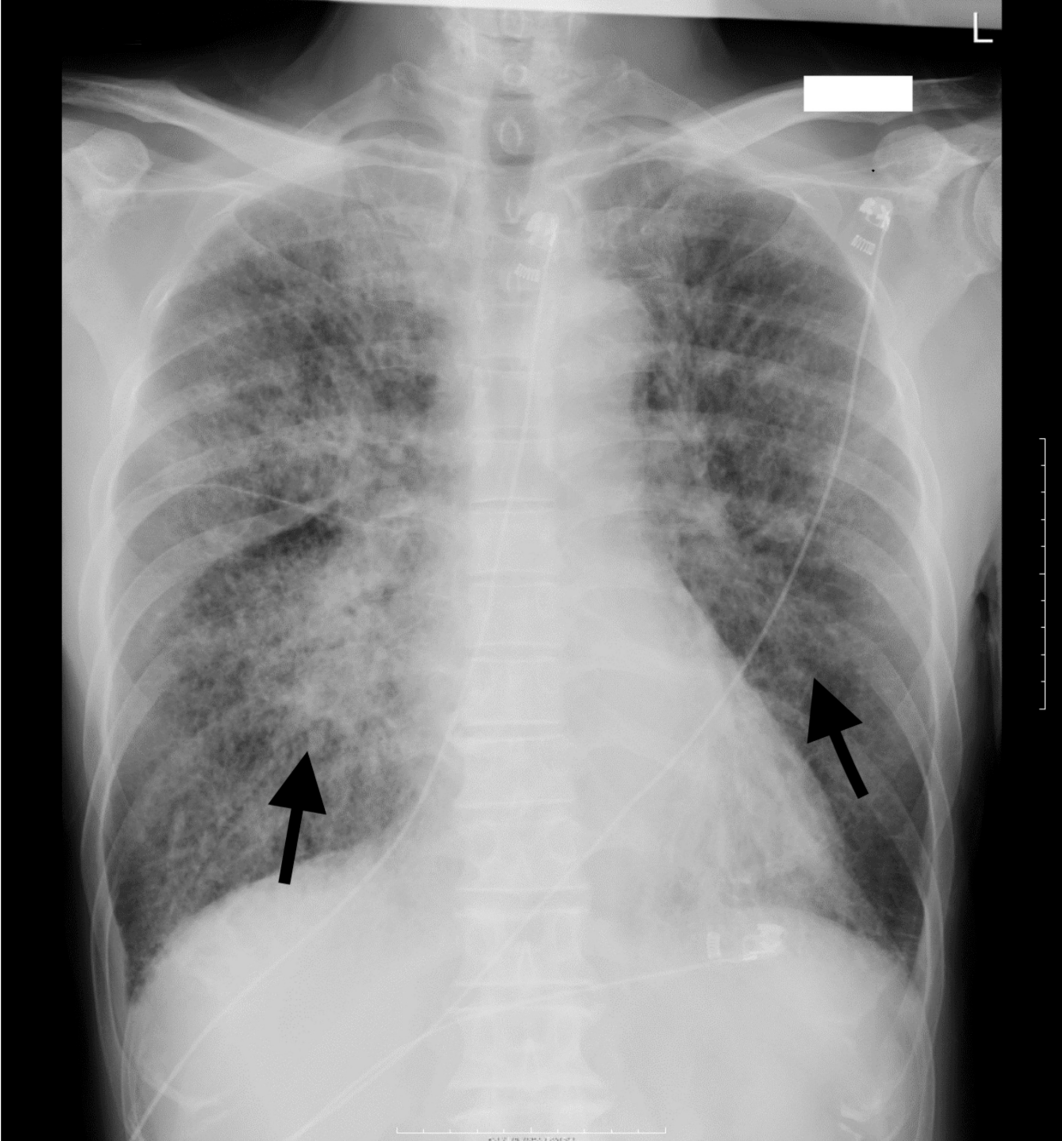
Chest radiography showed extensive frosted shadows in bilateral lung fields with no evidence of cardiac enlargement (cardiothoracic ratio, 48%).

Chest radiography revealed extensive frosted shadows in bilateral lung fields with no evidence of cardiac enlargement (cardiothoracic ratio, 48%) (Figure 1).

**Figure 2:**
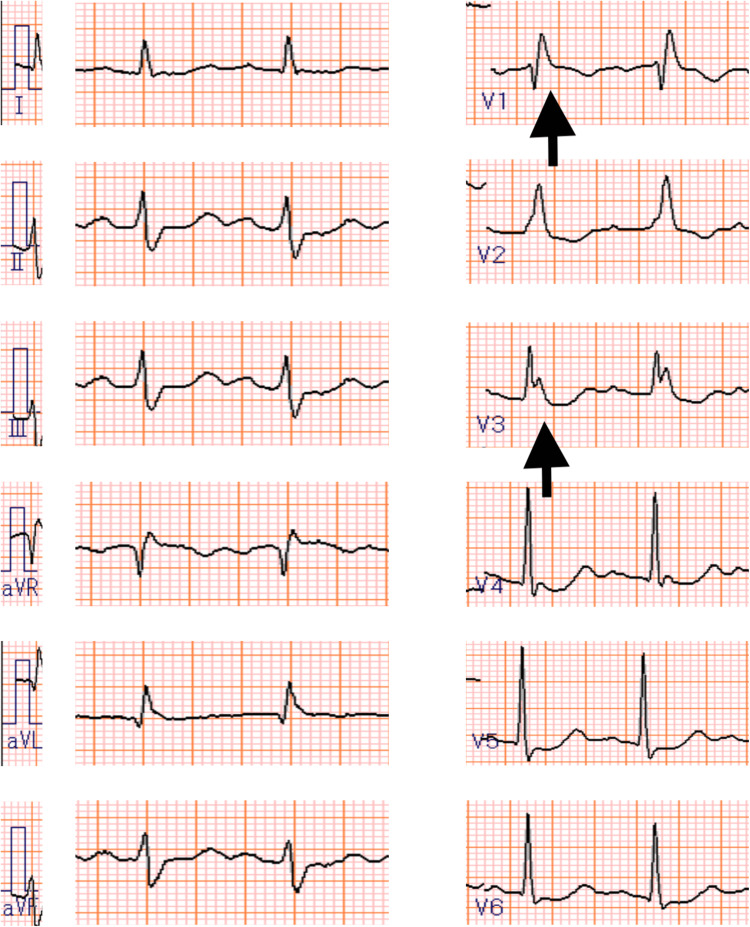
Electrocardiography showed no ST-segment changes and only a right bundle branch block.

Electrocardiography showed no ST-segment changes and only a right bundle branch block (Figure 2).

Transthoracic echocardiography (TTE) showed a hyperdynamic left ventricle with an ejection fraction (EF) of 70% and no loss of wall motion, valve regurgitation, cardiac enlargement, pericardial effusion, or right heart stress findings. The patient developed severe cardiorespiratory failure and was intubated. Computed tomography showed air bronchogram-positive equivocal shadows in all lung fields, mainly in the lower lung fields (Video 1).

**Video 1:** Computed tomography showed air bronchogram-positive equivocal shadows in all lung fields, mainly in the lower lung fields.

Based on the acute onset and imaging findings, severe pneumonia was suspected, and broad-spectrum antimicrobial therapy was initiated. Subsequently, bronchoscopy was performed; only pink, foamy secretions were observed, and there was no purulent sputum. Chest auscultation revealed a low systolic murmur without dissipation (Levine grade 2), strongest at the apex, and a clearly audible S2. Considering the possibility of cardiogenic shock from acute coronary syndrome, coronary angiography was performed; no stenosis was found in the coronary arteries (Videos 2-3).

**Video 2:** No stenosis was found in the coronary angiography of the left coronary artery.

**Video 3:** No stenosis was found in the coronary angiography of the right coronary artery.

Left ventriculography was performed, and mitral regurgitation (Seller’s classification Ⅳ) was observed (Video 4).

**Video 4:** Left ventriculography showed mitral regurgitation (Seller’s classification Ⅳ).

The patient was diagnosed as having heart failure due to acute MR. He was admitted to the intensive care unit and underwent mitral valve replacement on the fifth hospital day. Intraoperative findings showed thickening of the P2 and P3 segments of the mitral valve, with several torn chordae, but no vegetations. The intra-aortic balloon pump was removed on the sixth hospital day. The patient was extubated on the seventh day, transferred to the general ward on the ninth day, and discharged on the 28^th^ day. He is currently able to walk to the outpatient clinic.

## Discussion

Herein, we report a case of acute heart failure caused by acute MR, which can easily be misdiagnosed. Zhou et al. reported that 60% of patients are misdiagnosed at the time of initial treatment, with some reportedly taking up to four days to be diagnosed. Two hundred and sixty-two consecutive echocardiograms with severe mitral regurgitation performed between February 2005 and October 2010 at the Jack D. Weiler Hospital (Bronx, New York, USA) were reviewed, and 15 patients were found to have acute flail mitral valve. A mitral regurgitant murmur was appreciated in only a third of the patients. Clinically, 60% were misdiagnosed on admission. Using an echocardiogram, the correct diagnosis of the flail mitral valve was made in all cases; however, only 40% were on the day of presentation [4]. The reasons for misdiagnosis include difficulty in obtaining characteristic findings on physical and echocardiographic examinations, and the onset of the disease may be acute rather than sudden [2]. Chest auscultation often fails to reveal a heart murmur, and echocardiography fails to capture the reflex jet. The heart remains less compliant in acute and severe MR than in chronic MR. The left atrium has no time to expand, left atrial compliance remains low, and left atrial pressure rises rapidly towards late systole owing to high backflow into the left atrium [3-6]. As a result, the pressure gradient between the left atrium and ventricle rapidly decreases, and the duration of regurgitation is shortened. This results in an inaudible heart murmur and the reflux is not captured by echocardiography.

In cases of acute pulmonary edema and moderate-to-severe acute MR, a heart murmur has been reported to be inaudible in 87% of cases with normal systolic function, 68% of cases with reduced systolic function, and 74% overall [7]. However, a phonocardiogram is characteristic. In chronic MR, S2 is difficult to hear, and a high-pitched pansystolic murmur is heard; however, in the present case, S2 was clearly heard, and a low-pitched, diamond-shaped, progressively decreasing murmur was heard. This may be due to a rapid increase in left atrial pressure and a rapid decrease in left ventricular pressure, resulting in a decrease in the pressure gradient towards the end of systole and a reduction in the heart murmur [1, 8]. The principle of heart sounds is that the higher the gradient, the higher the pitch, and the higher the flow, the lower the frequency [6].

Although some studies are reporting that echocardiography may not detect a regurgitant jet in acute MR [8], none using quantitative data could be found. A TTE may reveal low blood volume from the left ventricle to the aorta despite normal to hypercontractile wall motion without cardiac enlargement, which may be suspicious for MR. Stroke volume can be approximated by measuring the area of the aortic EF (velocity-time integral (VTI) or the blood flow rate through the aortic valve; a VTI below 15 suggests decreased stroke volume [9]. 

There are two possible modes of disease onset: sudden and acute. In sudden onset, complete rupture occurs instantaneously. In acute onset, partial tears of the chordae spread sequentially over a wide area, or the normal chordae are subjected to greater stress at certain sites, resulting in chordal rupture [2]. In the present case, the latter mechanism is thought to have caused chordal rupture. Ischemic is the most common cause, followed by nonischemic. Embarrassingly, in this case, at the time of valve replacement, the specimen was not submitted to pathology, and we were unable to reach a definitive histological diagnosis. However, since there was no coronary artery stenosis on coronary angiography, ischemia was ruled out. Blood culture was negative; infective endocarditis (IE) was ruled out. Collagen disease was unlikely based on medical history. Intraoperative findings of mucous tumor changes and age-related changes suggested myxomatous degeneration as the cause.

Acute MR, particularly when presenting with cardiogenic unilateral pulmonary edema, is often misdiagnosed as pneumonia. Due to retropapillary obstruction, the regurgitant jet is directed towards the right superior pulmonary vein opening; an infiltrating shadow first appears localized to the right superior lobe, followed by expansion of the shadow to the other lung fields and contralateral lung. A particularly high rate of misdiagnosis has been reported in patients with unilateral cardiogenic pulmonary edema [10].

Cardiovascular diseases (CVDs) are the primary cause of death worldwide and significantly diminish the quality of life. Delayed diagnosis in acute MR can lead to death. Clinicians should bear in mind the fact that misdiagnosis of acute MR is not infrequent [11].

## Conclusions

Heart failure caused by acute MR may have atypical findings during physical examination and TTE, which can complicate its diagnosis. Although auscultatory listening for a murmur and echocardiographic detection of reflux waves are important findings in the diagnosis of acute MR, these findings may not be obtained depending on the time since onset. Healthcare providers must maintain a high index of suspicion for acute heart failure in patients with respiratory distress and hemodynamic instability. Early identification of valvular abnormalities can significantly improve outcomes. Raising awareness of acute MR presentation and complications is essential for emergency care teams to ensure appropriate patient care.
